# Phylogeography of the marine pathogen, *Vibrio vulnificus*, revealed the ancestral scenarios of its evolution

**DOI:** 10.1002/mbo3.1103

**Published:** 2020-08-10

**Authors:** Naiel Bisharat, Yael Koton, James D. Oliver

**Affiliations:** ^1^ Department of Medicine D Emek Medical Center Clalit Health Services Afula Israel; ^2^ Ruth and Bruce Rappaport Faculty of Medicine Israel Institute of Technology‐Technion Haifa Israel; ^3^ Department of Biological Sciences The University of North Carolina at Charlotte Charlotte NC USA

**Keywords:** ancestral character reconstruction, maximum likelihood, phylogenetics, phylogeography, *Vibrio vulnificus*

## Abstract

*Vibrio vulnificus* is the leading cause of seafood‐associated deaths worldwide. Despite the growing knowledge about the population structure of *V*.* vulnificus*, the evolutionary history and the ancestral relationships of strains isolated from various regions around the world have not been determined. Using the largest collection of sequence and isolate data of *V*.* vulnificus* to date, we applied ancestral character reconstruction to study the phylogeography of *V*.* vulnificus*. Multilocus sequence typing data from 10 housekeeping genes were used for the inference of ancestral states and reconstruction of the evolutionary history. The findings showed that the common ancestor of all *V*.* vulnificus* populations originated from East Asia, and later evolved into two main clusters that spread with time and eventually evolved into distinct populations in different parts of the world. While we found no meaningful insights concerning the evolution of *V*.* vulnificus* populations in the Middle East; however, we were able to reconstruct the ancestral scenarios of its evolution in East Asia, North America, and Western Europe.

## Graphical abstract

Using a global collection of multilocus sequence typing data from *Vibrio vulnificus* spanning over 55 years, this study provides insights into the spatial and temporal dynamics of the species evolution. The common ancestor of all *V. vulnificus* populations originated in East Asia, and later evolved into two main clusters that spread with time and eventually evolved into distinct populations in North America and Western Europe.

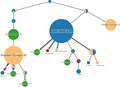

## 1 INTRODUCTION

*Vibrio vulnificus* is a naturally occurring inhabitant of estuarine and marine environments throughout the world and resides in high numbers in filter‐feeding shellfish (oysters, clams, and mussels) (Oliver, 2006). This bacterium is responsible for the vast majority of seafood‐related deaths worldwide (Oliver, 2006), usually resulting from the ingestion of raw oysters. More frequently, human disease is caused by direct contact of an open wound with seawater, leading to wound infection. Immunocompromised patients are at the highest risk of developing severe complications. Chronic liver diseases have been considered the factor that most predisposes for catastrophic complications (Haq & Dayal, 2005). Also, malignancy, end‐stage renal disease, chronic immunosuppressive therapy, and iron‐overload associated conditions such as hemochromatosis and thalassemia are all associated with increased susceptibility to *V*.* vulnificus* infection (Oliver, 2006). The global epidemiology of *V*.* vulnificus* has only emerged relatively recently. Since its recognition as a pathogen to humans in the 1970s, disease cases were largely reported from the USA and East Asia (Chuang, Yuan, Liu, Lan, & Huang, 1992; Hlady, Mullen, & Hopkins, 1993; Klontz et al., 1988). However, human infections have been progressively reported from many parts of the world (Strom & Paranjpye, 2000). The emergence of *V*.* vulnificus* in Israel and Western Europe was attributed, in part, to climate change and rising seawater temperatures (Baker‐Austin, Trinanes, Gonzalez‐Escalona, & Martinez‐Urtaza, 2016; Paz, Bisharat, Paz, Kidar, & Cohen, 2007; Sterk, Schets, de Roda Husman, de Nijs, & Schijven, 2015). The phylogenomic analysis showed that *V*.* vulnificus* populations are largely divided into two main groups and two small clusters, one cluster dominated by strains from Western Europe and the other cluster by strains from Israel (Koton, Gordon, Chalifa‐Caspi, & Bisharat, 2015; Lopez‐Perez et al., 2019; Roig et al., 2018).

Despite the growing knowledge about the population structure of *V*.* vulnificus*, the evolutionary history and the ancestral relationship of strains isolated from Western Europe and Israel with those from other parts of the world have not been determined. Ancestral character reconstruction (ACR) can be used to recover various ancestral character states, including the genetic sequence (ancestral sequence reconstruction), protein sequence, gene order, morphological properties, and the geographical range of an ancestral population or species (Joy, Liang, McCloskey, Nguyen, & Poon, 2016; Pagel, Meade, & Barker, 2004; Pupko, Pe'er, Shamir, & Graur, 2000). Molecular sequences collected in present time provide abundant information about past evolutionary events. Relevant probabilistic models of molecular evolution enable reconstructing ancestral sequences to a sample of taxa, and phylogenetics provides an adequate framework for the reconstruction of ancestral sequences. From a phylogenetic tree that depicts the evolutionary history of a sample of taxa, along with a set of corresponding homologous sequences, the sequences at each internal node of the tree can be estimated (Oliva et al., 2019). ACR is commonly used to reconstruct ancestral sequences that correspond to specific tree nodes. This can also enable determining changes in a character of interest on a phylogenetic tree over evolutionary time, by assigning the most likely ancestral character states to every internal node (Ishikawa, Zhukova, Iwasaki, & Gascuel, 2019). Global reconstruction over the entire tree describes the ancestral scenario of the character of interest.

In this study, we applied a phylogeography framework to reconstruct the spatial and temporal dynamics of *V*.* vulnificus* based on strains isolated from various geographical areas around the world.

## 2 MATERIALS AND METHODS

### 2.1 Bacterial strains data

We used sequence data from 10 housekeeping genes that comprise the multilocus sequence typing (MLST) scheme of *V*.* vulnificus*. The MLST scheme of *V*.* vulnificus* includes hundreds of sequences and data of isolates submitted to https://pubmlst.org by researchers during the past 15 years (2004–2019). The MLST scheme of *V*.* vulnificus* was devised by one of the authors (NB) (Bisharat et al., 2005) and is currently hosted at https://pubmlst.org/vvulnificus/. Also, we extracted MLST sequence data from draft or complete genomes of 133 strains available at https://www.ncbi.nlm.nih.gov. The extraction of MLST sequence data and the assignment of alleles and sequence types were carried out using MLST 2.0 (Larsen et al., 2012), which is available at https://cge.cbs.dtu.dk/services/MLST/.

As of September 2019, data of sequences and isolates of a total of 707 strains were available for this study. These included 1260 alleles of 10 MLST loci, resulting in 530 unique sequence types (ST). Of the 707 strains, 219 were obtained from human tissues and the remaining (*n* = 488) from environmental sources (water and sediment), fish or eel, and shellfish. The list of strains used in this study and their assigned STs and alleles are available in Tables A1 and A2 (https://doi.org/10.6084/m9.figshare.12666104).

### 2.2 Phylogeny, phylogeography, and ancestral character reconstruction (ACR)

We used 530 concatenated sequences from 10 housekeeping genes that comprise the MLST scheme of *V*.* vulnificus*. Maximum‐likelihood (ML) phylogenies were inferred using the rapid tree searching approach implemented in PhyML (version 3.0) (Guindon et al., 2010), using general time reversal (GTR) with automatic model selection by SMS (smart model selection) (Lefort, Longueville, & Gascuel, 2017). Subsequently, the phylogenetic tree with the ML method was analyzed with IQ‐TREE (Nguyen, Schmidt, von Haeseler, & Minh, 2014) using the default settings, with 1000 bootstrap values for tree evaluation. IQ‐TREE (http://www.iqtree.org/) calculates and maps the likelihood of all possible sequence quartets using the best‐fitting nucleotide substitution model (Schmidt, Strimmer, Vingron, & von Haeseler, 2002). GrapeTree was used to visualize the phylogenetic relationships between the various isolates. GrapeTree is a web‐browser application that efficiently reconstructs and visualizes intricate minimum spanning trees, together with detailed metadata (Zhou et al., 2018). For the phylogeographic studies, this dataset was annotated with: sampling years (between 1964 and 2018); countries, which we grouped into 7 regions (North America, East Asia, Southeastern Asia, Western Europe, Middle East, South America, and Australia); and genotypes representing all three existing biotypes (biotypes 1, 2, and 3). We inferred an ML tree from the DNA sequences and rooted the tree using *Vibrio parahemolyticus* as an outgroup. The phylogeography of *V*.* vulnificus* was reconstructed from the ML tree, and locations were annotated using PastML with default options (Ishikawa et al., 2019). PastML uses decision‐theory concepts to associate each node in the tree with a set of likely states. In the easy regions of the tree (typically close to the tips), the program predicts a unique state, whereas, in the more challenging parts of the tree (typically close to the root), it may predict several likely states, reflecting the uncertainty of the inferences. PastML takes as input a rooted tree (as described above) and a tip state annotation table. It produces a table with predicted ancestral states and interactively modifiable visualization. The full tree was later uploaded and annotated with the ancestral predictions to Interactive Tree of Life (ITOL), an interactive tool for tree management and visualization (Letunic & Bork, 2016). Since large phylogenies with hundreds of tips are difficult to visualize and interpret, PastML provides a compressed representation of the ancestral scenarios while highlighting the main information and hiding minor details. The default options for running PastML in the current study were as follows: the marginal posterior probability approximation (MPPA) method and the F81 model, which provides the most accurate ancestral predictions (see the detailed description in Ishikawa et al., 2019). PastML enables prediction of the ancestral scenario at the root and subroot, and along the tree up to the tips, employing two approaches used in maximum likelihood‐based ACR: the maximum a posteriori (MAP) and the joint ancestral scenario (Joint). The MAP is done by selecting the state with the highest posterior from all the marginal posterior probabilities of every state for each of the tree nodes (Yang, 2007). The Joint is computed with the maximal posterior probability using dynamic programming (Pupko et al., 2000).

## 3 RESULTS

The most frequent ST in the dataset was ST8, representing 69 strains isolated from Israel, of which 95.6% were isolated from human clinical samples. Other common STs included ST112 (representing strains from human and non‐human samples from Western Europe and Australia), ST139 and ST140 (representing strains isolated from eels in Western Europe), ST6 (representing strains isolated from eels in East Asia), ST136 (representing environmental strains from North America), and ST32 (representing strains isolated from human clinical samples in North America). No single ST was predominant among strains isolated from human clinical samples in East Asia.

### 3.1 Phylogenetic analysis

**FIGURE 1:**
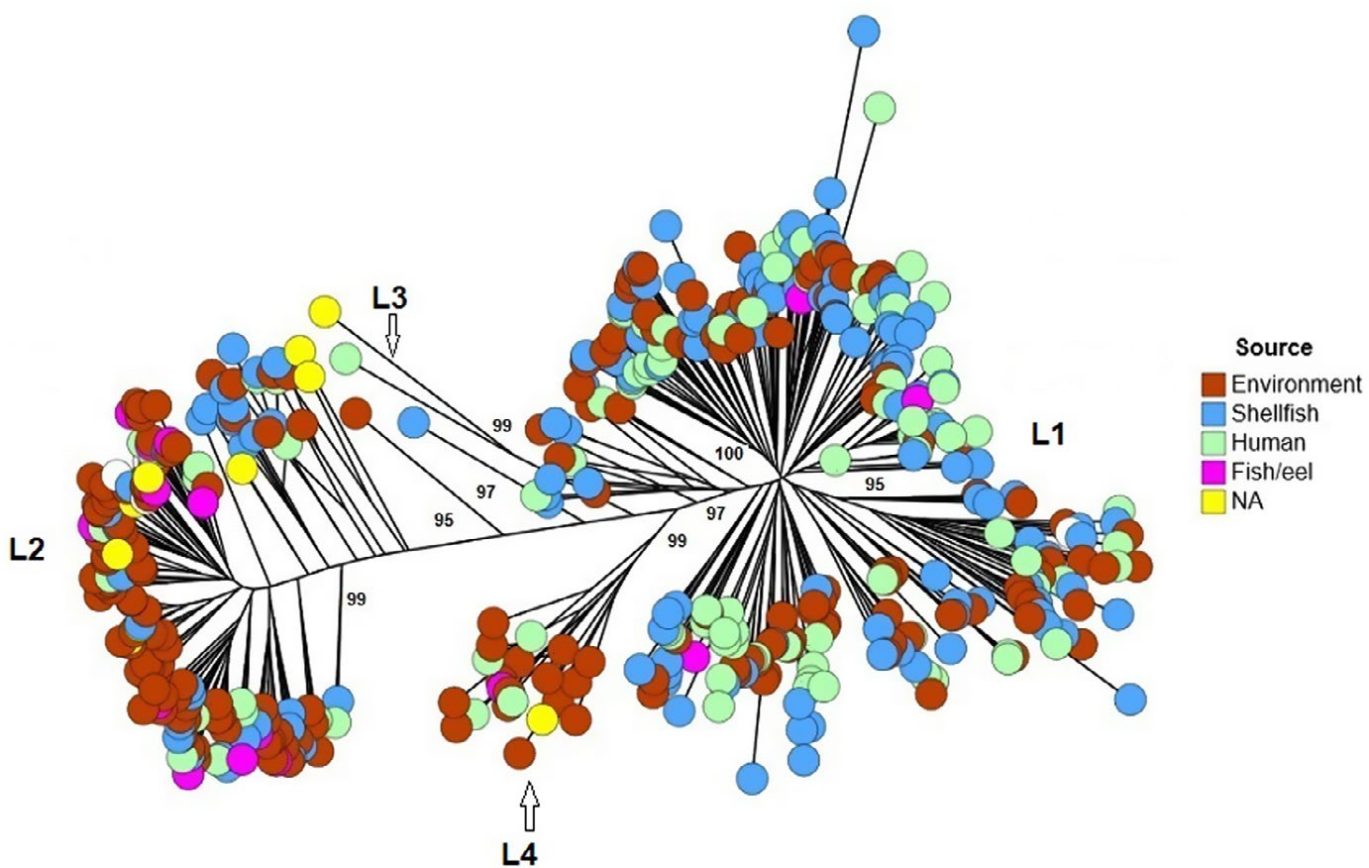
Phylogeny and population structure of *Vibrio vulnificus* produced by GrapeTree, based on isolates' source. Four distinct lineages are shown. Lineage I is dominated by human‐pathogenic strains, lineage II is dominated by environmental strains, and lineage III consists of biotype 3 strains (all resolved into one genotype). Bootstrapping was carried out with 1000 replicates. Branch node values below 50% are not shown. For clarity reasons, not all bootstrap values are shown

**FIGURE 2:**
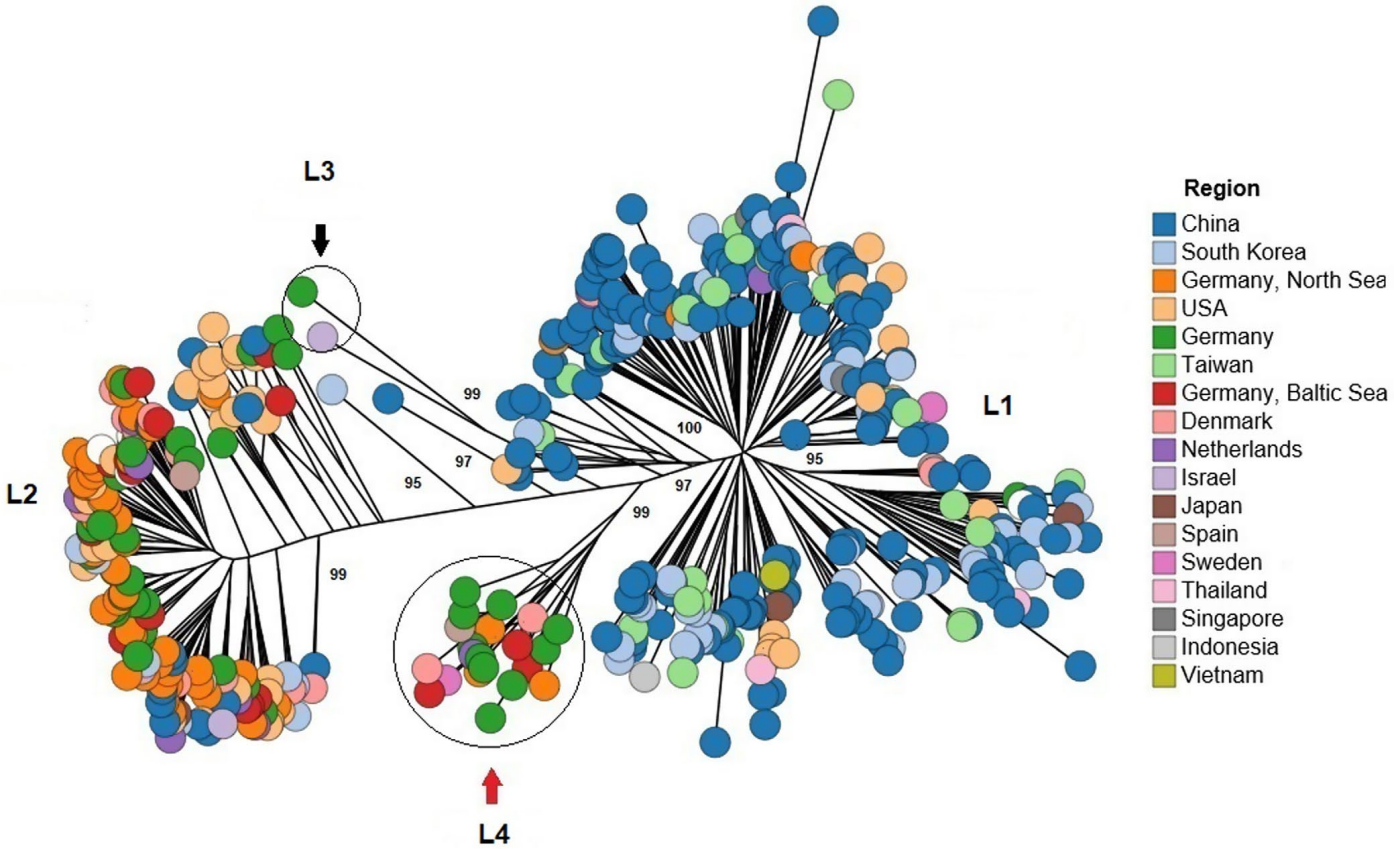
Phylogeny and population structure of *Vibrio vulnificus* based on the geographical source of the isolates. Lineage III consists of strains originating almost entirely from Israel, and lineage IV consists of strains from Western Europe. Bootstrapping was carried out with 1000 replicates. Branch node values below 50% are not shown. For clarity reasons, not all bootstrap values are shown

Phylogenetic analysis showed that *V*.* vulnificus* populations are divided into two main lineages, both of which included environmental and human‐pathogenic strains. One lineage (lineage I) was dominated by strains from human samples, while another (lineage II) was dominated by strains from environmental sources (Figure 1). Two other small distinct lineages were also identified, lineage III consisting of biotype 3 strains (all belonging to sequence type 8) and lineage IV dominated by environmental strains. The same analysis based on the geographical source showed that the vast majority of strains within lineage I was dominated by strains from East and Southeastern Asia, while lineage II strains mainly originated from Germany, USA, and the Netherlands (Figure 2). Lineage III consisted of biotype 3 strains from Israel and a closely related genotype from Western Europe. Lineage IV consisted of strains originating from Western Europe (Germany, Spain, Denmark, and the Netherlands). The list of strains with their assigned STs and lineages is shown in Table A1 (https://doi.org/10.6084/m9.figshare.12666104).

### 3.2 Phylogeography and ancestral sequence reconstruction

**FIGURE 3:**
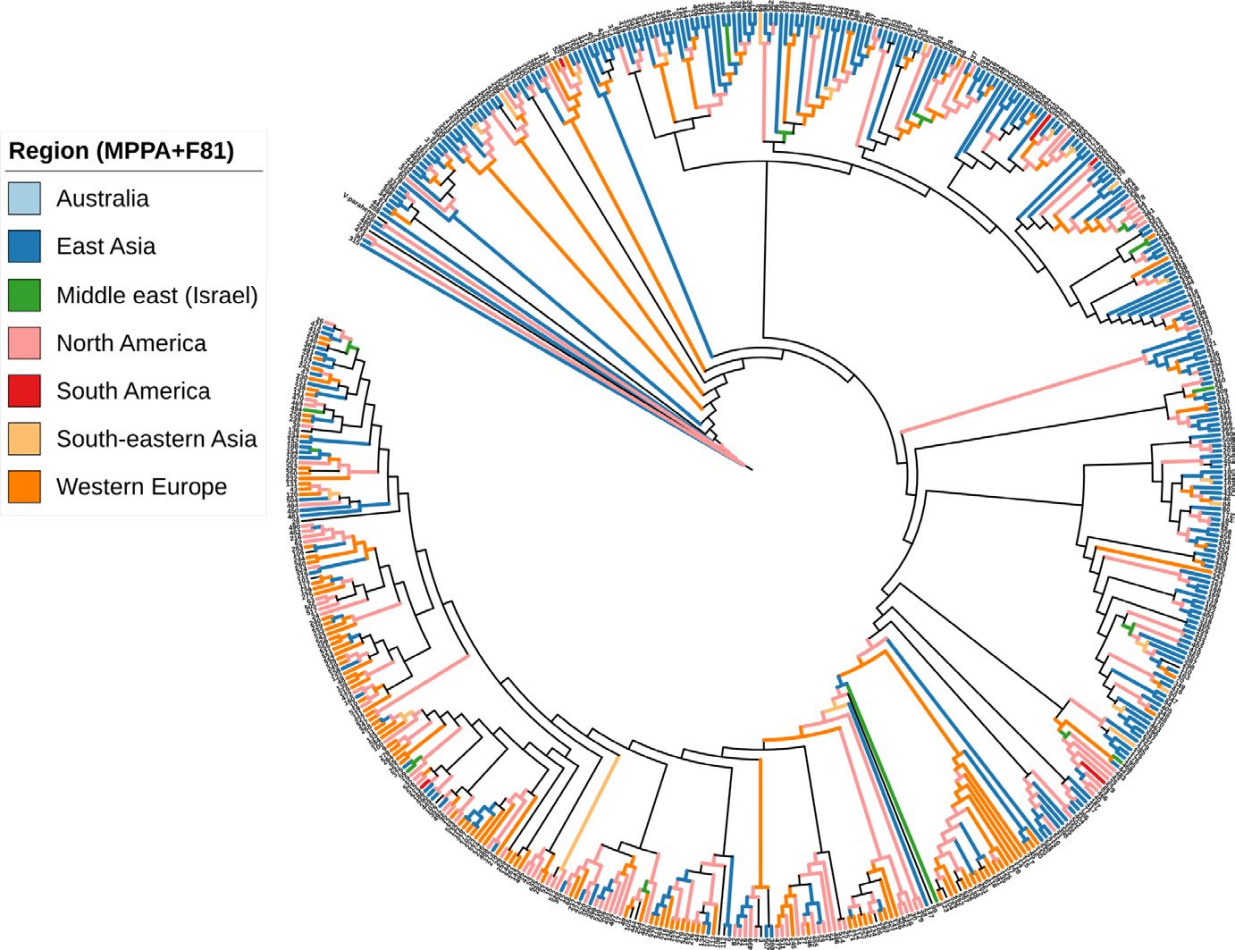
ITOL presentation of the ancestral reconstruction of *Vibrio vulnificus* locations based on the full visualization mode of PastML using marginal posterior probability approximation (MPPA) with an F81‐like model. Different colors correspond to different geographical regions. The numbers at the outer circle indicate sequence types

**FIGURE 4:**
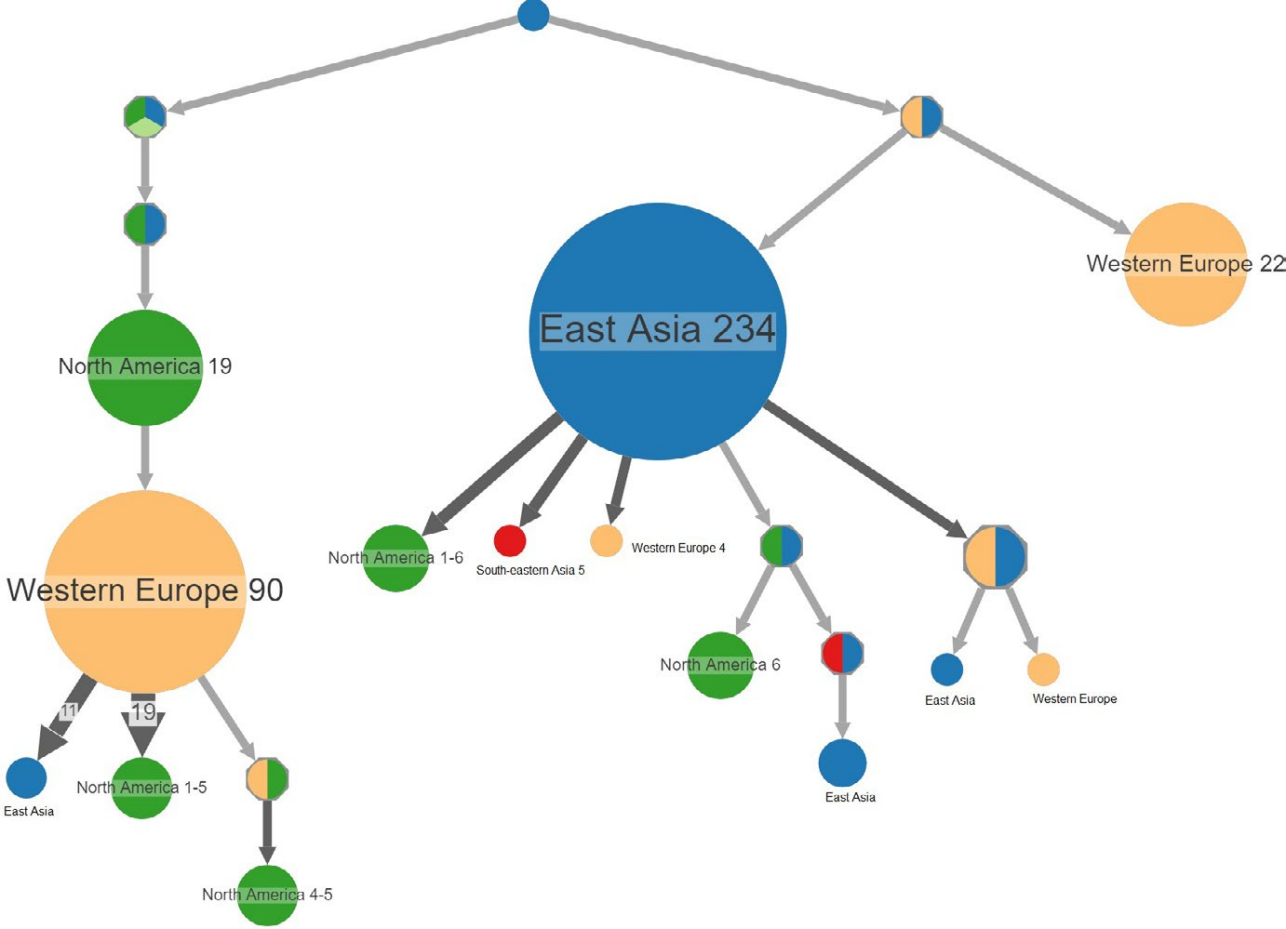
Ancestral reconstruction of *Vibrio vulnificus* locations. The figure shows the compressed visualization produced by PastML using marginal posterior probability approximation (MPPA) with an F81‐like model. Different colors correspond to different geographical regions. The joint ancestral scenario (Joint) and maximum a posteriori (MAP) predictions are shown for the uncertain nodes (shown as octagonal icons). MAP and Joint are discrepant regarding the root of the left and right subtrees, but their predictions are included in MPPA predictions for all the nodes. Numbers inside (or next to) the circles indicate the number of strains assigned to the specific node

PastML results based on the output of the PhyML tree are shown in Figure 3. This depicts a full visualization tree produced by ITOL, with the likely ancestral scenarios at each node of the tree. A compressed image is shown in Figure 4. The location of the root is in East Asia, which represents the most likely common ancestor for all the populations of *V*.* vulnificus*. Along the tree, there are nodes (shown as octagonal icons) where PastML could not accurately predict the ancestral state due to a discrepancy between the two main approaches used in the maximum likelihood‐based ACR: the MAP and the Joint. From the root, two possible ancestral states emerge, right and left subtrees. The right subtree root could not be resolved, but from there, the main East Asian cluster emerges (shown as a large blue circle representing 234 strains) (Figure 4), and a Western European cluster with 22 strains. From the largest East Asian cluster, multiple introductions occurred, leading to smaller clusters in North America, Southeastern Asia, and Western Europe. The left subtree root could not be resolved, but from there, a North American cluster emerges that spreads to become the largest cluster from Western Europe (representing 90 strains). From this point, multiple introductions occurred, leading to smaller clusters in North America and East Asia. The combined ancestral states for every part of the tree are shown in Table A3 (https://doi.org/10.6084/m9.figshare.12666104). The accuracy of MPPA predictions at the various nodes was remarkable, as determined by the average number of states per node ~1.05. Of nearly 1060 nodes, only 61 were unresolved. The log‐likelihood of the MAP, Joint, and MPPA scenarios was equal to −1246.4, −1238.9, and −1218.7, respectively. As expected, Joint was better than MAP, and MPPA was even better, as it includes several states for some of the nodes.

## 4 DISCUSSION

Ancestral sequence reconstruction, using the combination of phylogenetic trees with extrinsic traits, was used to decipher the evolutionary scenarios of *V*.* vulnificus*. This study showed that the common ancestor of all *V*.* vulnificus* populations most likely originated in East Asia, and later evolved into two main clusters that spread and eventually evolved into distinct clusters in East Asia, North America, and Western Europe.

Despite the availability of powerful methods and software in ancestral character state inference, interpreting the outputs of such inferences may be challenging. The approach implemented in the current study minimized the uncertainty of ancestral inferences, especially in the difficult regions of the tree. This ultimately represented a larger fraction of the data while producing a scenario that is almost fully resolved.

Previous studies established the division of *V*.* vulnificus* populations into two main lineages; lineage I, also known as C clade (enriched for clinical strains), and lineage II, also known as E clade (enriched for environmental strains) (Bisharat et al., 2005; Cohen, Oliver, DePaola, Feil, & Boyd, 2007; Lopez‐Perez et al., 2019; Roig et al., 2018; Rosche, Yano, & Oliver, 2005). The current study provided new insights into the evolution of these populations and determined its phylogeography. The validity of the methodology used in the current study was tested on two distinct datasets (Ishikawa et al., 2019).

The vast majority of strains isolated in East Asia shared the same ancestral origin, while strains isolated from the USA had more than one ancestral origin. Strains isolated from Western Europe were represented in both subtrees. The largest cluster of Western European populations descended from North American ancestry, while the other cluster evolved from East Asian ancestry.

This study did not result in meaningful insights concerning the evolution of *V*.* vulnificus* populations in the Middle East (Israel). This may be a result of poor sampling of strains in Israel. From 74 strains, 68 were isolated from human clinical samples and they all resolved into one human‐pathogenic genotype, ST8. The remaining 6 strains, which were isolated from fish and water samples, displayed 5 distinct genotypes, represented in the two subtrees. More sampling of *V*.* vulnificus* from environmental sources is required to determine the ancestral scenarios of its populations in the Middle East. Nevertheless, *V*.* vulnificus* biotype 3, which is geographically restricted to Israel and is responsible for nearly all clinical cases of *V*.* vulnificus* in Israel, is likely to have evolved from biotype 1 populations (Bisharat et al., 2005, 2007; Danin‐Poleg, Elgavish, Raz, Efimov, & Kashi, 2013; Efimov et al., 2014; Raz et al., 2014). Given the genetic relatedness between strains from Israel and Western Europe, it is reasonable to postulate that they share the same ancestral origins.

The ancestral scenarios for the evolution of *V*.* vulnificus* suggest an East Asian common ancestor. However, the earliest reports of human infection come from the USA in the mid‐1970s (Fernandez & Pankey, 1975; Hollis, Weaver, Baker, & Thornsberry, 1976) and later from East Asia during the mid‐1980s (Chan, Woo, Lo, & French, 1986; Chuang et al., 1992; Lee, Chung, & Lee, 2013; Park, Shon, & Joh, 1991). Human infections in Western Europe and the Middle East were reported during the 1990s (Bisharat et al., 1999; Bisharat & Raz, 1996; Bock et al., 1994; Dalsgaard, Frimodt‐Moller, Bruun, Hoi, & Larsen, 1996; Torres, Escobar, Lopez, Marco, & Pobo, 2002; Veenstra, Rietra, Coster, Slaats, & Dirks‐Go, 1994). The global epidemiology of *V*.* vulnificus* is complex, and the changes observed are likely to have been the result of multiple factors such as the prevalence of the organism in the environment (Faruque & Nair, 2006), the impact of global warming on seawater temperatures which is extending the geographical range of *V*.* vulnificus* into the Northern Hemisphere (Vezzulli et al., 2016), and the emergence of new virulent strains due to the high and frequent horizontal gene transfer in the *Vibrionaceae* (Efimov et al., 2014; Kim et al., 2011; Quirke, Reen, Claesson, & Boyd, 2006). It is possible that improved diagnostics of infectious diseases may have played a role in shaping the epidemiology of *V*.* vulnificus*.

Our findings were based on sequence data from 10 housekeeping genes supplemented by sampling locations and sampling dates. There are normally fewer polymorphic sites in individual housekeeping genes than in hypervariable genes. Nonetheless, the use of the combined sequences of multiple housekeeping genes has been shown to provide high discriminatory power while retaining signatures of long‐term evolutionary relationships (Feil & Enright, 2004; Margos et al., 2008). The phylogeny inferred from MLST data in this study was highly congruent with the phylogeny inferred from draft or complete genomes (Lopez‐Perez et al., 2019; Roig et al., 2018). This challenges the observations made by some authors that MLST‐based phylogeny is inaccurate compared with phylogeny inferred from concatenated blocks of sequences or genome single‐nucleotide polymorphism profiles (Tsang, Lee, Yiu, Lau, & Woo, 2017). The population structure and evolution of *V*.* vulnificus* share some interesting features with the most closely related vibrios, *Vibrio parahemolyticus* and *Vibrio cholerae*. Populations of *V*.* parahemolyticus* are subdivided into 4 distinct geographical populations, two of which appear to have foci in the USA, and a third is predominant in Asia. The ancestral home range of the forth (VppX), found in North America and Western Europe, remains unclear (Yang et al., 2019). Similarly, persistent aquatic environmental reservoirs for *V*.* cholerae* O1 are present in Asia, causing a disease that has recurrently manifested in seven worldwide pandemics originating from Asia (Colwell & Huq, 1994; Mavian et al., 2020; Weill et al., 2017).

This study has some limitations that should be acknowledged when interpreting the findings. First, our conclusions regarding the ancestral states and the reconstruction of the evolutionary history of this species were based on sequences of 10 housekeeping genes and not whole‐genome sequences, which has become a standard approach for studying the population structure and dynamics of bacteria. Within this context, using sequences of a limited number of conserved genes could be considered an acceptable proxy for inferring the population structure and evolution as has been recently used for studying the evolution of other vibrios (Moore et al., 2015; Ono et al., 2019). Furthermore, the resolution provided by MLST can be considered acceptable since sequence data included in the MLST scheme provide a valid phylogenetic signal (as described above) to infer the phylogeography and reconstruction of ancestral states. Finally, one of the most important aspects in studies aiming to define the population structure, phylogeography, or routes of dispersal is the coverage (in space and time) of the pool of strains included in the study. To obtain reliable results, it is critical to use collections with a wide geographical and temporal coverage. For this aim, the historical repository of data deposited on the MLST website is a unique source of sequence data, in terms of a number of strains, countries of origin, and long time span. Second, another limitation of the study is the possible unbalance in the composition of the database with a dominance of clinical strains for some lineages (e.g., the Israeli strains that are dominated by clinical isolates). The absence of representative strains from environmental sources may introduce some bias and hence provide a partial picture of the genomic landscape of *V*.* vulnificus*. This may also imply that some human‐pathogenic strains may have been imported via marine products from other sites. We believe that this scenario (import through the marine product) is highly unlikely in the case of disease emergence in Israel. Lineage 3 clinical strains (Israel) have not been isolated in any other part of the world and have evolved from existing non‐pathogenic environmental populations (Bisharat et al., 2005). Third, the methodology applied in this study for the inference of ancestral states and reconstruction of the evolutionary history is based on ML phylogeny. This methodology was initially tested on viruses with short sequences and rapid evolution (over short periods, few months to years) (Korber et al., 2000; Yang, Lauder, & Lin, 1995). Within this context, ML approaches effectively estimate the mutation rate and reconstruct ancestral scenarios. However, ML methodologies are not equally effective for bacteria, detecting less ancestral states (nodes in the tree) than other approaches based on Bayesian inference. Nevertheless, and rather interestingly ML‐based methodology has been recently used for studying the evolution of *Vibrio cholerae* reservoirs in aquatic ecosystems (Mavian et al., 2020).

## 5 CONCLUSIONS

Using a global collection of MLST sequence data of *V*.* vulnificus* spanning over 55 years, this study provided insights into the spatial and temporal dynamics of the evolution of *V*.* vulnificus*. We urge investigators of *V*.* vulnificus* to submit MLST data to PubMLST.org and thus contribute further knowledge and insights to the evolution of this important marine pathogen.

## CONFLICT OF INTEREST

None declared.

## AUTHOR CONTRIBUTIONS

**Naiel Bisharat:** Conceptualization (lead); data curation (lead); formal analysis (lead); investigation (lead); methodology (lead); project administration (lead); software (lead); supervision (lead); visualization (lead); writing – review & editing (lead). **Yael Koton:** Data curation (supporting); software (equal); writing – original draft (equal). **James D. Oliver:** Validation (supporting); writing – review & editing (supporting).

## ETHICS STATEMENT

None required.

## Data Availability

The datasets generated and analyzed during the current study are available at https://pubmlst.org/vvulnificus. Tables A1‐A3 are available in figshare: https://doi.org/10.6084/m9.figshare.12666104.
